# Editorial: Ion channels, pumps, and transporters in lens physiology and disease

**DOI:** 10.3389/fphys.2022.1071215

**Published:** 2022-11-03

**Authors:** Eric C. Beyer, Viviana M. Berthoud, Julie C. Lim, Paul J. Donaldson

**Affiliations:** ^1^ Department of Pediatrics, University of Chicago, Chicago, IL, United States; ^2^ Department of Physiology, New Zealand National Eye Centre, Faculty of Medical and Health Sciences, The University of Auckland, Auckland, New Zealand

**Keywords:** lens, ion channel, connexin, aquaporin, transporter, cataract, water channel

The lens is a transparent organ located near the front of the eye whose major function is to project an undistorted image of incoming light onto the retina. To achieve this the lens must maintain its transparency and refractive properties over many decades of life and in the process it needs to overcome some unique physiological challenges that are not experienced by other tissues. Being a large avascular organ that is suspended by the zonular fibers between the aqueous and the vitreous humors, the lens must exchange its nutrients and waste products without the assistance of a blood supply. The major bulk of the lens is formed by fiber cells that lack organelles; they are covered by an anterior layer of epithelial cells which differentiate at the lens equator to form the fiber cells. The degradation of nuclei and organelles that occur during normal epithelial cell-to-lens fiber cell differentiation contribute to the minimization of light scattering (reviewed in (Bassnett et al., 2011)). To compensate for the absence of a blood supply and the paucity of cellular organelles, the lens operates an internal microcirculation system that uses circulating ion and water fluxes to maintain cellular homeostasis and therefore the transparency and refractive properties of the lens at the whole tissue level (Mathias et al., 2007). These circulating ion and water fluxes are generated by spatial differences in an extensive repertoire of ion channels, pumps and exchangers that drive ion and water movement throughout the lens (Gao et al., 2011; Vaghefi et al., 2011; Candia et al., 2012) to facilitate the delivery of nutrients to the center of the lens (Vaghefi and Donaldson, 2018) and the active regulation of the transparency and refractive properties of the lens (Vaghefi et al., 2015). Because the lens channel and transport proteins are critical components of its circulation system, it is now becoming apparent that their dysfunction can cause changes to the optical properties of the lens that manifest as presbyopia in middle age and cataracts in the elderly (Donaldson et al., 2017).

This Research Topic covers several different areas of lens biology. These articles and reviews consider various lens ion channels and transporters and their regulation, post-translational modifications, alterations by mutations in other protein genes, and complex inter-relationships. Together, these papers help to elucidate the normal and pathological state of the lens microcirculation, lens cell homeostasis and maintenance of lens transparency.

Giannone et al. provide a concise update on the lens circulation model, giving some consideration to the effects of oxidation and aging on the lens circulation and their impact on vision. Beyer et al. review the alterations in components of the lens microcirculation reported in various studies of different mouse cataract models. From these studies, they conclude that disruption of intercellular communication between fiber cells is a common feature to many of these models, even in the absence of mutations in the connexin genes. Retamal and Altenberg focus their review on gap junction channels and hemi-channels composed of connexin46 and how their properties and regulation are affected by different post-translational modifications. Some of these modifications may contribute to the changes in lens intercellular communication associated with aging and cataracts.

Giannone et al. also highlight recent studies showing that forces transmitted through the zonules (which would represent a mechanical stimulus) can lead to changes in the hydrostatic pressure gradient. Interestingly, Ebihara et al. considered the possibility that fluid flow in and out of individual lens cells (as it would occur with shape changes during accommodation) are modulated by pressure-activated channels. The results of their patch-clamp studies implicate activation of calcium-activated chloride channels by mechanical stimulation, a process that may involve influx of extracellular calcium through TRPV4 channels. Delamere and Shahidullah review the recent findings regarding the roles of TRPV1 and TRPV4 channels in the activation of different signaling pathways in the lens. The TRPV4 feedback loop senses lens swelling and leads to an increase in Na^+^, K^+^-ATPase activity, while the TRPV1 feedback loop senses shrinkage and leads to an increase in the activity of the Na^+^/K^+^/2Cl^−^ cotransporter, NKCC1.

The survival of the organ in the absence of a blood supply and of its cells devoid of organelles continue to be intriguing issues. Further insights into the handling of glucose in the lens are reported in the article by Zahraei et al. They use stable isotope labeling and mass spectrometry to examine the patterns of glucose uptake and subsequent metabolism in bovine lenses. They conclude that the major site of glucose uptake is at the lens equator, and they correlate their findings with the distributions of different glucose transporters. Water channels are also differentially distributed in the lens. In their review, Schey et al. describe how the differential distribution, water permeability and regulation of three water channels or aquaporins (AQP0, AQP1, and AQP5) and the changes in their subcellular localization in the different lens regions contribute to lens water transport. The authors incorporate these recent findings to propose an updated model of the lens microcirculation system.

Many studies have linked mutations in different genes (including connexin46, connexin50, AQP0, various crystallins, transcription factors, and other lens proteins) to human cataracts (https://cat-map.wustl.edu/) (Shiels et al., 2010). Although in many cases the mechanism by which these mutations lead to disease is unknown, it is reasonable to speculate that they might lead to alterations of ion transport in the lens. In their article, Cheng et al. have extended our knowledge regarding the importance of Eph-ephrin signaling. They find that disruptions of EphA2 and ephrin-A5 in the lens lead to alterations of the distributions of connexin50 and aquaporin0 and hyperpolarization of fiber cell membranes. However, no differences in gap junctional coupling were detected in EphA2- or ephrin-A5-null mice.

Overall, this Research Topic has brought together contributions that address some contemporary issues in lens biology and physiopathology and provide a critical appraisal of significant historical advances in this research area.
